# Balance Evaluation Based on Walking Experiments with Exoskeleton Interference

**DOI:** 10.3390/bioengineering11040386

**Published:** 2024-04-16

**Authors:** Liping Wang, Xin Li, Yiying Peng, Jianda Han, Juanjuan Zhang

**Affiliations:** 1 Tianjin Key Laboratory of Intelligent Robotics, Institute of Robotics and Automatic Information System, Nankai University, Tianjin 300350, China; lipingwang@mail.nankai.edu.cn (L.W.); 2120190365@mail.nankai.edu.cn (X.L.); yiyingpeng@mail.nankai.edu.cn (Y.P.); hanjianda@nankai.edu.cn (J.H.); 2 College of Artificial Intelligence, Nankai University, Tianjin 300350, China; 3 School of Materials Science and Engineering, Smart Sensing Interdisciplinary Science Center, Nankai University, Tianjin 300350, China

**Keywords:** walking balance, statistical analysis, principal component analysis, ankle exoskeleton

## Abstract

The impairment of walking balance function seriously affects human health and will lead to a significantly increased risk of falling. It is important to assess and improve the walking balance of humans. However, existing evaluation methods for human walking balance are relatively subjective, and the selected metrics lack effectiveness and comprehensiveness. We present a method to construct a comprehensive evaluation index of human walking balance. We used it to generate personal and general indexes. We first pre-selected some preliminary metrics of walking balance based on theoretical analysis. Seven healthy subjects walked with exoskeleton interference on a treadmill at 1.25 m/s while their ground reaction force information and kinematic data were recorded. One subject with Charcot–Marie–Tooth walked at multiple speeds without the exoskeleton while the same data were collected. Then, we picked a number of effective evaluation metrics based on statistical analysis. We finally constructed the Walking Balance Index (WBI) by combining multiple metrics using principal component analysis. The WBI can distinguish walking balance among different subjects and gait conditions, which verifies the effectiveness of our method in evaluating human walking balance. This method can be used to evaluate and further improve the walking balance of humans in subsequent simulations and experiments.

## 1. Introduction

Walking balance has always been a concern in the field of medical rehabilitation. Impaired walking balance function seriously affects human health, especially for patients and the elderly with mobility difficulties. The human body can use fast and powerful muscle contraction and relaxation to move joints quickly to maintain posture and balance. The strength of muscles is critical for providing the power to regulate balance [1]. Decreased muscle strength, slowed muscle response, and limited range of motion of joints all result in the ineffective implementation of balance strategies, which affects human locomotion ability [2], disturbs the stability of posture [3], and eventually leads to frequent falls [4]. Falling can easily lead to a variety of health problems, such as fracture, joint deformity, dislocation, and soft tissue injury [5]. Therefore, the walking balance of humans, especially those with limited mobility, is a matter of concern. It is crucial to provide assistance for them and improve their walking balance. We first need to evaluate the state of human walking balance and aim to optimize it in future works.

The evaluation methods for balance are usually grouped into clinical tests, scaling methods, and quantitative measurements by instruments. Clinical tests, such as the Romberg Test [6,7], are easy to apply but inaccurate and subjective. These methods are only suitable for the preliminary screening of patients with suspected balance problems [8]. Scaling methods include the Berg Balance Scale [9], Tinetti Test [10], and so on. Compared to clinical tests, scaling methods are quantified by levels and are relatively more accurate. However, the scaling methods still have a certain subjectivity [11,12]. The determination of grading boundaries depends on the personal judgment of evaluators [13,14]. Quantitative measurement methods, such as the pressure plate method [15], have the advantage of being able to accurately measure the state of balance. Existing quantitative measurement methods select metrics only based on the intuition and experience of professionals and cover fewer metrics.

Researchers have used a lot of metrics to evaluate balance. The commonly selected metrics are the human body’s center of mass (COM) [4,16,17] and the trajectory of the center of pressure (COP) [18,19,20]. When the human is standing still, the body’s stability can be evaluated by the position of the COM [16]. Whether an additional step is required to avoid a fall can also be obtained by analyzing the position and the velocity of the COM [21]. In static balance, measuring the swing trajectory of the COP with a force plate has become a standard for evaluating static balance ability [18,22]. Additionally, it has shown that the distance and velocity between the anterior and lateral displacements of the COP contribute to explaining the walking balance [19,20]. Acceleration information from human body can also be used to assess human walking balance [23,24]. The Lyapunov index of the COP’s speed and the trunk acceleration of the elderly who are prone to falling are significantly higher than those of normal people [24]. Experts put forward the stability criterion of the Zero Moment Point (ZMP), which provides an effective solution for the balanced judgment of human walking [25]. The relative position between the centroidal moment pivot (CMP) and ZMP can supplement information about the rotational dynamics of the human body. In this study, we considered the COP and ZMP as the same points [26] so we replaced the position of the ZMP with the position of the COP. In addition, the margin of stability (MOS) contains information on position and velocity which can describe changes in human motion [21,27]. However, a single metric is not effective or comprehensive as it does not consider or reflect all factors influencing balance during human locomotion. It is necessary to combine multiple metrics and establish a comprehensive quantitative index to evaluate the walking balance of different individuals under different walking conditions.

The purpose of this study was to present a method to construct an evaluation index of human walking balance that (1) comprehensively selects more preliminary metrics related to walking balance in theory, (2) reduces human factors through being driven by pure data, and (3) can quantify and evaluate human walking balance in a way that can distinguish between different individuals under different walking conditions. We collected gait data from eight subjects, seven healthy subjects and one Charcot–Marie–Tooth (CMT) patient. The main clinical symptoms for patients with CMT, like muscle weakness and atrophy, seriously affect their walking balance. Then, we used our method to generate personal and general indexes. We verified the effectiveness of this method in evaluating human walking balance and analyzed the main influencing metrics of balance during human walking. This study is expected to provide guidance for future research on walking balance evaluation, explore the potential factors that influence human walking balance, and further improve the walking balance function for the required population in the future.

## 2. Methods

We pre-selected a list of preliminary metrics for human walking balance through theoretical analysis. Then, we recruited eight subjects, seven healthy subjects and one subject with CMT. We conducted walking experiments with exoskeleton interference to collect data for the selected metrics from the seven healthy subjects. We also collected the same data from the CMT subject at multiple speeds. We further picked subsets of these metrics that significantly deviated between different gait conditions through statistical analysis. We eventually constructed a comprehensive quantitative evaluation index of walking balance by combining multiple metrics using principal component analysis (PCA). We computed and analyzed the results of our method to verify its effectiveness in evaluating human walking balance.

### 2.1. Experimental Platform

**Figure 1:**
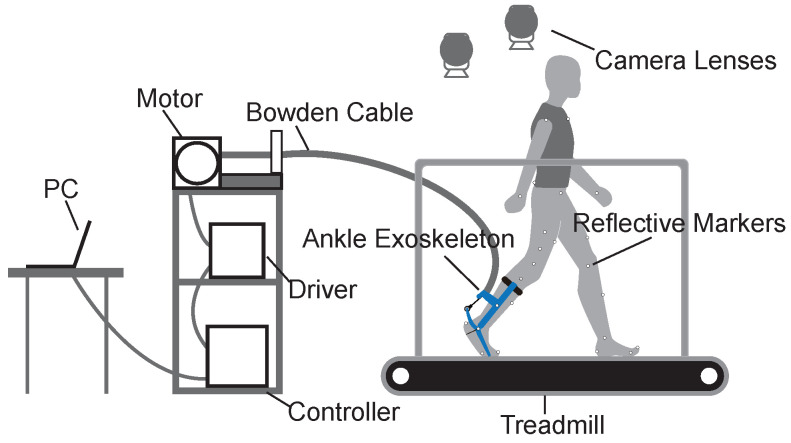
Gait experimental platform. The gait experimental platform included a control system, an actuation system, transmissions, a motion capture system, and an ankle exoskeleton.

We used a gait experimental platform to collect data on the preliminary metrics of human walking balance (Figure 1). The experimental platform consisted of a force-measuring treadmill, an optical motion capture system, and an ankle exoskeleton system. The ankle exoskeleton system was driven by an off-board actuation system with a Bowden cable and controlled by a real-time control (DS1202, dSPACE, Paderborn, GmbH) system.

#### 2.1.1. Treadmill

We used the gait analysis treadmill (Bertec, Columbus, OH, USA) in this study. The treadmill can measure forces and moments in three directions of an orthogonal coordinate system. Some related balance metrics, such as the positions of the COP, can be calculated by measuring the real-time ground reaction forces and moments.

#### 2.1.2. Motion Capture System

We used an optical motion capture system (Oqus 700+, Qualisys, Sweden) with ten high-speed camera lenses at a sampling rate of 100 Hz to accurately catch the motion of subjects with reflective markers. The basic motion data of subjects, such as the velocities and accelerations of limbs, can be calculated through analysis software.

#### 2.1.3. Ankle Exoskeleton System

We used the ankle exoskeleton to apply ankle interference torques and to obtain relatively unbalanced gait data. The ankle exoskeleton is mainly composed of three components: a calf frame, a foot frame, and a forefoot frame. The interference torque is transmitted through a Bowden cable and applied at the ankle joint. The concrete structure of the ankle exoskeleton can be found in the previous article [28].

The ankle exoskeleton uses several sensors to measure human–robot interactions. A magnetic encoder (PQY 18, ACCNT, Dongguan, China) was fixed on the lateral shaft of the exoskeleton to measure ankle joint angles. A load cell (DYMH-106, DAYSENSOR, Bengbu, China) was seated at the ankle lever to measure Bowden cable force. A footswitch (KW12, Risym, Shenzhen, China) was installed on the heel of the shoe to detect heel strike during gait cycles.

#### 2.1.4. Control and Actuation System

The control and actuation system were mounted on a shelf next to the treadmill. We used the real-time control system (DS1202, dSPACE, Paderborn, Germany) to sample data from sensors at 5000 Hz and drive the actuation system at 500 Hz. The actuation system was composed of an AC servo motor, a 5:1 planetary gear, and a motor driver (BSM90N-175AA, GBSM90-MRP120-5, and MF180-04AN-16A, ABB, Zurich, Switzerland).

### 2.2. Pre-Selection of Preliminary Metrics

In accordance with previous studies, we pre-selected the following 14 balance metrics related to human walking balance:

Group A, the positions and velocities of the COP:

(1) $COP_{x}$, the position of the COP on the sagittal axis;

(2) $V_{COP_{x}}$, the velocity of the COP on the sagittal axis;

(3) $COP_{y}$, the position of the COP on the coronal axis;

(4) $V_{COP_{y}}$, the velocity of the COP on the coronal axis.

Group B, the accelerations of the COM:

(5) $A_{COM_{x}}$, the acceleration of the COM on the sagittal axis;

(6) $A_{COM_{y}}$, the acceleration of the COM on the coronal axis;

(7) $A_{COM_{z}}$, the acceleration of the COM on the vertical axis;

(8) $A_{COM}$, the resultant acceleration of the COM.

Group C, the positions of the COM:

(9) $COM_{x}$, the position of the COM on the sagittal axis;

(10) $COM_{y}$, the position of the COM on the coronal axis;

(11) $COM_{z}$, the position of the COM on the vertical axis.

Group D, other metrics:

(12) COP_CMP, the distance between the CMP and COP;

(13) MOS, the MOS of the trunk;

(14) $A_{ANG}$, the angular acceleration of the trunk.

The real-time positions of the COP can be calculated by the ground reaction forces and moments as follows:

(1) $$\begin{array}{c} COP_{x}=\frac{-h\cdot F_{ground,x}-M_{ground,y}}{F_{ground,z}} \end{array}$$

(2) $$\begin{array}{c} COP_{y}=\frac{h\cdot F_{ground,y}-M_{ground,x}}{F_{ground,z}} \end{array}$$

where $F_{ground,x}$, $F_{ground,y}$, and $F_{ground,z}$ are the components of the ground reaction forces along the three coordinate axes, respectively. $M_{ground,x}$ and $M_{ground,y}$ are the ground reaction moments in the sagittal and coronal plane, and *h* is the height difference between the belt and the x–y plane of the treadmill coordinate system.

The velocities of the COP can be obtained by:

(3) $$\begin{array}{c} V_{COP_{x}}=\frac{d}{dt}COP_{x},V_{COP_{y}}=\frac{d}{dt}COP_{y} \end{array}$$

The position of the human body’s COM can be expressed by:

(4) $$\begin{array}{c} COM=\sum_{i=1}^{n}\frac{m_{i}\cdot com_{i}}{M} \end{array}$$

where $com_{i}$ and $m_{i}$, respectively, represent the centroid position and mass of the *i*th segment of the human body, *M* represents the total mass of the human body, and *n* is the number of human body segments.

The accelerations of the COM can be obtained by:

(5) $$\begin{array}{c} A_{COM_{x}}=\frac{d^{2}}{dt^{2}}COM_{x},A_{COM_{y}}=\frac{d^{2}}{dt^{2}}COM_{y},A_{COM_{z}}=\frac{d^{2}}{dt^{2}}COM_{z}, \end{array}$$

(6) $$\begin{array}{c} A_{COM}=\sqrt{A_{COM_{x}}^{2}+A_{COM_{y}}^{2}+A_{COM_{z}}^{2}} \end{array}$$

The position of the CMP can be calculated from ground reaction forces and the position of the COM as follows:

(7) $$\begin{array}{c} CMP_{x}=COM_{x}-\frac{F_{ground,x}}{F_{ground,z}}\cdot COM_{Z} \end{array}$$

(8) $$\begin{array}{c} CMP_{y}=COM_{y}-\frac{F_{ground,y}}{F_{ground,z}}\cdot COM_{Z} \end{array}$$

The MOS proposed by Terry et al. [27] was adopted in this study. The MOS can be expressed as:

(9) $$\begin{array}{c} MOS=\left|\left(COP+\frac{V_{COP}}{\omega_{0}}\right)-\left(COM+\frac{V_{COM}}{\omega_{0}}\right)\right| \end{array}$$

(10) $$\begin{array}{c} \omega_{0}=\sqrt{\frac{g}{l}} \end{array}$$

where *g* is the acceleration of gravity, and *l* is the height of the COM.

We also selected three time domain characteristics as the gait features for each metric above: root mean square (RMS), variance (Var), and range (Range). The RMS has a non-negative feature which is often used to characterize the effective value of the data. Var characterizes the degree of dispersion of the data distribution. Range reflects the range of the data, emphasizing the extreme values. The three selected time domain characteristics show different aspects of the collected data. Therefore, 14 balance metrics were selected, and three time domain characteristics were selected for each balance metric. A total of 42 preliminary metrics were selected in this study.

### 2.3. Experimental Protocol

Seven healthy subjects (five males and two females; age = 23.3 ± 1.0 years; body mass = 67.9 ± 12.4 kg; height = 175.4 ± 6.5 cm) participated in this study. We also recruited one male subject with CMT (age = 14 years; body mass = 35.3 kg; height = 160 cm) to verify the feasibility of our method for the patient (Table 1). The study was conducted according to the Helsinki Declaration and approved by the ethical committee of Nankai University (reference number: NKUIRB2021054). All subjects provided written informed consent before completing the protocol. All methods used in this study were performed in accordance with the relevant guidelines and regulations.

We designed and conducted walking experiments with exoskeleton interference based on the experimental platform to collect the preliminary metric data for the healthy subjects in relatively balanced and unbalanced gait conditions. The human ankle plays a vital role in balance, propulsion, and locomotion in a wide range of environments encountered. Two important mechanisms for balance control are the stepping strategy and the lateral ankle strategy. The ankle strategy is faster than the stepping strategy [29]. The timing, magnitude, and speed of torque produced by the ankle joint all affect walking balance [30,31,32]. Therefore, we used the ankle exoskeleton to apply ankle interference torque, affecting walking balance.

**Figure 2:**
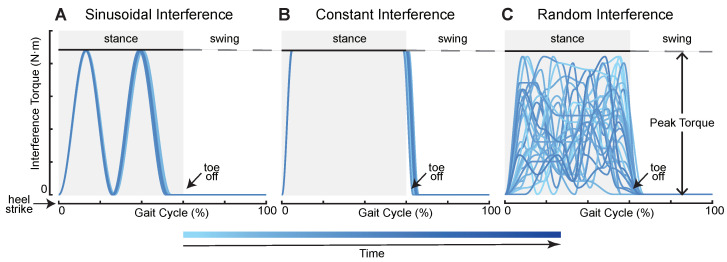
Three ankle interference torque profiles. These torques were applied only in the support phase (0–60% of a gait cycle) for safety. (**A**) Sinusoidal interference torque. It consisted of two continuous sinusoids. (**B**) Constant interference torque. Its maximum torque remained the same. (**C**) Random interference torque. The torque profile was generated randomly.

To create external interference for subjects during their walking, we first defined four interference torque profiles, sinusoidal interference torque (SIN), constant interference torque (CON), random interference torque (RAN), and mixed interference torque (MIX). The first three profiles are shown in Figure 2. SIN consisted of two positive continuous sinusoids which started at heel strike and ended before toe off. The SIN had two peak values that were given at the ankle joint mainly during the mid-stance and terminal stance phase. The CON was similar to a step signal whose maximum torque remained the same. We used two cubic splines to form the rise and fall phase of the CON. The RAN was generated by random combinations of sine and cosine signals. Its curves changed each stride, and peak values were applied to the ankle joint at any time during the stance phase. The MIX was a combination of the first three interference torque profiles whose order was SIN–CON–RAN–SIN–CON–RAN, and the duration of each application of interference torque was the same. We used two parameters to limit the generation of torque profiles: peak torque and applied time. Peak torque refers to the maximum allowable torque that the subjects could bear. The peak torque of each subject is shown in Table 1. Applied time refers to the time when the interference torque was applied to subjects from the first heel strike to the ground. For safety reasons, we only applied ankle interference in the supporting phase (0–60% of a gait cycle) during walking. The interference torque was tracked using a combination of proportional–derivative control and an iterative learning algorithm [33,34]. The effectiveness of this torque control method has been demonstrated in the previously published literature. The torque controller can be found in previous articles [35,36].

The walking experiments with exoskeleton interference for healthy subjects were divided into two parts: zero torque (ZT), where subjects walked with the exoskeleton but no torque applied, and ankle interference torque, where subjects walked with the exoskeleton applying the interference torque. The seven healthy subjects walked on the treadmill at 1.25 m/s and wore the right ankle exoskeleton. The first healthy subject walked under a 30 s ZT condition followed by a 6 min MIX condition with a 2 min break in between. The other six healthy subjects first performed under a ZT condition for 30 s and then a SIN condition for 2 min, a CON condition for 2 min, a RAN condition for 2 min, and, finally, a MIX condition for 6 min with a 2 min rest between each condition. The CMT subject walked without the ankle exoskeleton, which was called normal walking (NW), on the treadmill at multiple speeds, which he could accept. We collected his gait data for 30 s at 0.45 m/s, 0.55 m/s, and 0.65 m/s. After a period of rest, a second round of experiments was carried out in reverse order, and the gait data were collected again. For each subject, 38 reflective markers (six on each foot, eight on each calf and thigh, three on the pelvis, one on the chest, and three on each arm) were affixed to the whole body during all experiments. We collected the motion capture data and treadmill data during all the experiments. The collected experimental data were used in the construction and validation of the comprehensive index for human walking balance.

### 2.4. Construction of Walking Balance Index

We processed the experimental data from the eight subjects and obtained the data for the selected metrics. We picked subsets of these metrics using statistical analysis and constructed the index by combining multiple metrics using PCA. The data processing was conducted using OpenSim 4.1 [37,38] and MATLAB (MathWorks, Natick, MA, USA). The statistical analysis and PCA were conducted using MATLAB.

#### 2.4.1. Data Processing

We synchronized and collected the three-dimensional ground reaction forces and moments (1000 Hz) data and motion capture (100 Hz) data of the eight subjects using the real-time controller. We pre-processed the experimental data with low-pass filters and scaled the musculoskeletal model with the height and weight of the subjects. We performed inverse kinematics using the Rajagopal2015 model in OpenSim [39]. We then calculated the 14 aforementioned balance metrics using the positions of marks and the masses of the human body segments. We segmented the metric data according to the gait cycles. For each metric in each gait cycle, we calculated all three time domain characteristics and summarized them into three data sets. We finally obtained the data sets of the 42 selected preliminary metrics under each gait condition.

#### 2.4.2. Statistical Analysis

Some of these metrics may be ineffective and even interfere with the evaluation of walking balance. It was necessary to further distinguish the effective metrics with evaluation ability from the preliminary metrics using statistical analysis.

From the data processing, we obtained data sets for the 42 selected preliminary metrics under each gait condition. For each subject, we used the significance tests to test all the preliminary metrics data with exoskeleton interference (SIN, CON, RAN, and MIX) and the preliminary metrics data without exoskeleton interference (ZT) in pairs.

We first used the Lilliefors test to test the normality of these data and the Bartlett test to test their homogeneity of variance. According to the results of normality and variance homogeneity tests, we chose the t-test for the data conforming to the normality and variance homogeneity tests, the corrected t-test for the data conforming to the normality but not homogeneity of variance, and the rank-sum test for the other data. All the above were two-sided tests. Considering the sample size (42 metrics per stride), the significance level was set at α = 0.01. We finally picked subsets of the preliminary metrics that significantly deviated between different gait conditions.

#### 2.4.3. Principal Component Analysis

There are always some correlations between the effective metrics we picked above so these needed to be further extracted.

The principle of PCA is to recombine original variables into a new set of unrelated comprehensive variables. This method can transform high-dimensional problems into low-dimensional problems to reduce the correlations. The new variables formed by a linear combination of the initial variables are the principal components. These principal components are independent of each other and largely resistant to man-made interference. The principal component coefficients and eigenvalues are also generated only based on data. Therefore, we used PCA to reduce the correlations among these effective metrics and constructed the final index.

We first standardized and decentralized the data of the effective metrics picked above. For subjects with different heights and weights, the values of distance metrics were different in the relatively consistent gait conditions. The units and orders of magnitude of these metrics were also different. Therefore, all the distance metrics were scaled according to the height and weight of each subject, and all the metrics needed to be dimensionless. We used the Kaiser–Meyer–Olkin test to test whether these effective metrics were correlated and could be analyzed by PCA.

We then calculated the covariance matrix of the metric data and calculated the principal component coefficients and eigenvalues of the covariance matrix through PCA. The principal component coefficients were used to compose principal components, and the eigenvalues were used to calculate the weights of the principal components. We used the principal components whose cumulative contribution rate exceeded 85% to replace the original metrics and constructed the comprehensive quantitative index, which we called the Walking Balance Index (WBI). The WBI can be expressed as follows:

(11) $$\begin{array}{c} y_{i}=\sum_{j=1}^{m}w_{ij}x_{j} \end{array}$$

(12) $$\begin{array}{c} WBI=\sum_{i=1}^{n}\frac{\lambda_{i}}{\sqrt{\sum_{j=1}^{n}\lambda_{i}^{2}}}y_{i} \end{array}$$

where $x_{j}$ and $w_{ij}$ are the *j*th effective metric and the coefficient of $x_{j}$ in principal component *i*, and *m* is the number of effective metrics after statistical analysis. $y_{i}$ and $\lambda_{i}$ are the *i*th principal component and the eigenvalue of $y_{i}$, and *n* is the number of principal components that we used.

### 2.5. Validation

We collected gait data from the seven healthy subjects under five gait conditions: ZT, SIN, CON, RAN, and MIX. We also collected gait data from the CMT subject at three speeds. Each combination of these data with and without exoskeleton interference can be used to construct the index. All experimental data can be used as validation data and to calculate the value of the WBI.

We mainly used the gait data of the seven healthy subjects obtained under ZT and MIX conditions to construct the personal indexes and compute their values. We also used the gait data of one healthy subject obtained under ZT and MIX conditions to construct the general index, and we used the data of six healthy subjects obtained under multiple gait conditions as validation data to compute the values of the general index. The data of the CMT subject were used to compute the value of the WBI constructed from subject1’s gait data obtained under ZT and MIX conditions. In addition, we also used the data of the CMT subject and subject1 to construct the index and compared the potential correlations and differences in the balance metrics between the CMT patient and healthy individuals.

Meanwhile, we obtained four gait combinations by combining the condition without interference (ZT) and four conditions with interference (SIN, CON, RAN, MIX) in pairs for the healthy subjects. We constructed and calculated the WBI using all combinations so as to further explore the possible disadvantages of this method, the main factors affecting the walking balance of humans, and the consistencies and differences of strategies for adjusting walking balance for different individuals.

## 3. Results

**Figure 3:**
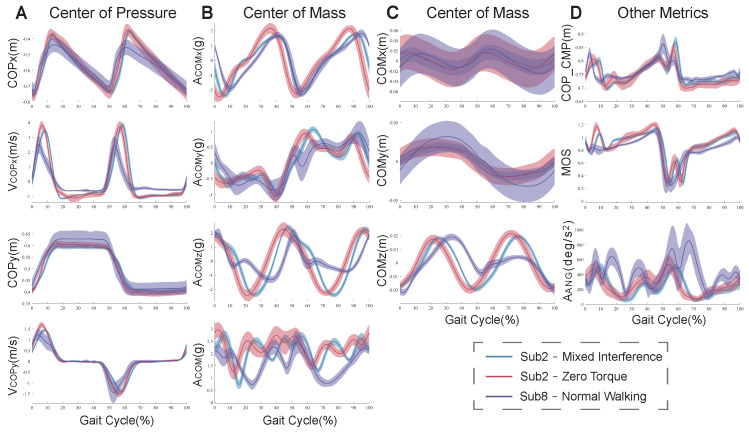
The changes in the preliminary metrics. (**A**) The changes in the positions and velocities of the COP. (**B**) The changes in the accelerations of the COM in three directions and the resultant acceleration. (**C**) The changes in the positions of the COM. (**D**) The changes in the relative position between the COP and CMP, MOS, and trunk angular acceleration. The blue curves show the changes for subject2 under the ZT condition. The red curves show the changes for subject2 under the MIX condition. The purple curves show the changes for subject8 with CMT under the NW condition. The shadow areas show the fluctuation range of different gait cycles.

Figure 3 shows the results of the preliminary metrics for the CMT patient (subject8) under the NW condition and healthy subject2 under ZT and MIX conditions. Compared to under the ZT condition, there were significant differences in these metrics under the NW condition for subject8, but there were fewer differences in them under the MIX conditions for subject2. The fluctuation range of the CMT patient was much larger than that of the healthy subject.

**Figure 4:**
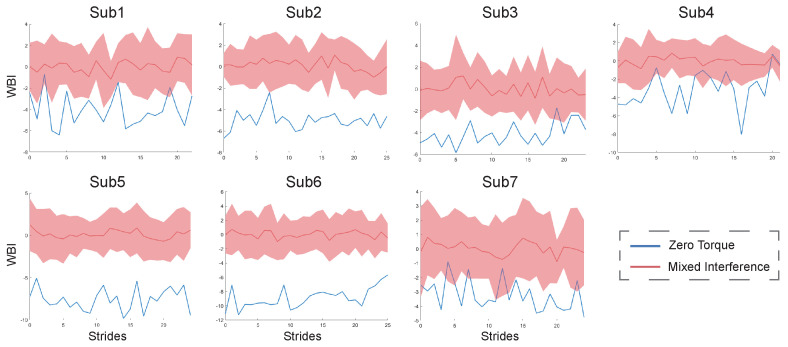
The values of personal WBI for seven healthy subjects. The blue curves are the WBI of each stride under the ZT condition. The red curves and areas are the mean and fluctuation range of the WBI under the MIX condition.

**Figure 5:**
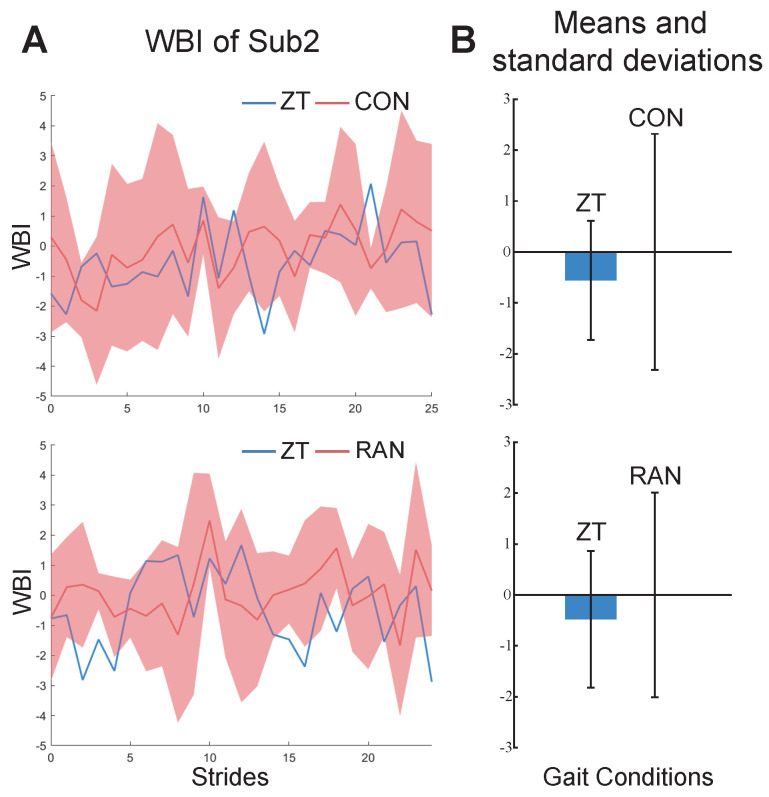
The results of WBI for subject2. (**A**) The WBI under ZT and CON conditions (above). The WBI under ZT and RAN conditions (below). (**B**) Bars and whiskers are means and standard deviations of the WBI for subject2 under ZT and CON conditions (above) and ZT and RAN conditions (below).

**Figure 6:**
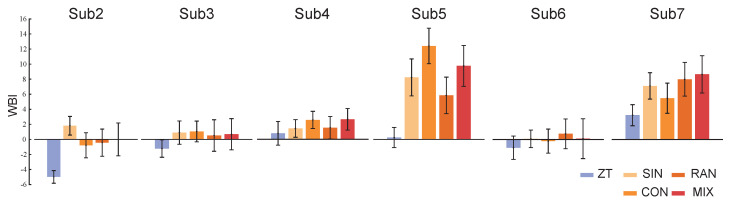
The values of the WBI for the six healthy subjects using the general index constructed from one subject’s data obtained under ZT and MIX conditions. Bars and whiskers are the means and standard deviations of the WBI for six healthy subjects under different gait conditions.

The results of the personal indexes for the seven healthy subjects under ZT and MIX conditions are shown in Figure 4. Compared to under the ZT condition, the values of their personal indexes under MIX conditions are bigger and have a wider range of fluctuations. The results of the constructed indexes under two gait combinations for subject2 are shown in Figure 5. The values of the WBI under the two combinations are too similar to be distinguished (Figure 5A). The means and standard deviations of the indexes under the ZT condition are still smaller than those under CON and RAN conditions (Figure 5B). The general index constructed from one of the healthy subjects under ZT and MIX conditions was used to calculate the means and standard deviations for the six healthy subjects under multiple gait conditions (Figure 6). The mean of the general index for each subject under the ZT condition was lower than that under other conditions.

Table 2 shows the results of the statistical analysis for subject1 under ZT and MIX conditions. A total of 25 effective metrics were picked from 42 preliminary metrics. They showed significant differences under the two different gait conditions. There was no significant difference in the information for $COP_{x}$, COP_CMP, or $A_{{COM}_{x}}$ (*p* > 0.01, Table 2). There were significant differences in the characteristics from the three time domains, $COP_{y}$, $V_{COP}$, $A_{{COM}_{y}}$, $A_{{COM}_{z}}$, and $COM_{z}$ (*p* < 0.01, Table 2). Other balance metrics showed significant differences in partial time domain characteristics. Table 3 shows the number of times that the metrics showed significant differences under four gait combinations for the six healthy subjects.

The results of the PCA for subject1 are shown in Table 4. Table 5 shows the coefficient matrix of principal components for subject1. The contributions of the first three principal components were more than 10% (Table 4). The $V_{COP}$, $COP_{y}$, $COM_{z}$, $A_{COM_{z}}$, and MOS had larger coefficients of principal components compared to the other metrics (Table 4 and Table 5).

**Figure 7:**
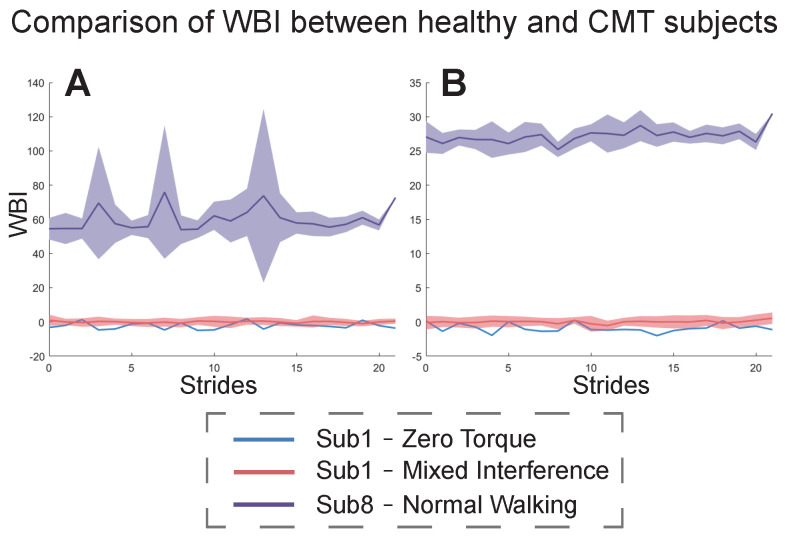
WBI of healthy subject1 under ZT and MIX conditions and subject8 with CMT under the NW condition. (**A**) The results of the WBI constructed from subject8’s NW condition and subject1’s MIX condition. (**B**) The results of the WBI constructed from subject1’s ZT and MIX conditions. The blue curves show subject1’s WBI for each stride under the ZT condition. The red curves and areas show the mean and fluctuation range of subject1’s WBI under the MIX condition. The purple curves and areas show the mean and fluctuation range of subject8’s WBI under the NW condition.

Figure 7 shows the results of the WBI for the CMT patient (subject8) under the NW condition with respect to subject1 under ZT and MIX conditions. The index in Figure 7A was constructed from subject8’s NW condition and subject1’s MIX condition. The index in Figure 7B was constructed from subject1’s ZT and MIX conditions. In both cases, the mean and fluctuation range of the CMT subject’s WBI was much larger than that of the healthy subject.

## 4. Discussion

This study presents a method to construct a comprehensive evaluation index of human walking balance, and we used this method to generate personal and general indexes. We designed walking experiments with exoskeleton interference to collect data from seven healthy subjects and collected data from one CMT subject at multiple speeds. We used statistical analysis and PCA to construct the Walking Balance Index. We verified the feasibility of this method in evaluating walking balance for healthy individuals and one CMT patient.

Healthy subjects were not significantly affected by the ankle interference torque (Figure 3). This may be because the applied torque was small (for safety). Due to the adjustment mechanism of human ankles, the effect of interference torque on one ankle joint is not obvious, and the impact on other parts of the human body is not great either. The fluctuation range of these metrics increased under the MIX condition with respect to the ZT condition, indicating that interference torque can affect the amplitude of human motion. Especially the metrics related to COM were affected more obviously. When human lower limbs are disturbed, the human will unconsciously adjust the trunk’s posture to keep the COM within the support surface. Studies have shown that insufficient trunk muscle strength affects the balance ability of humans and increases the risk of falling [4]. The CMT subject’s metrics fluctuated more widely with respect to those of the healthy subject with exoskeleton interference, indicating that the state of walking balance for the CMT subject is worse than that for the healthy subject. The walking strategies of patients are different from that of healthy individuals. The ankle movements of CMT patients are limited due to the muscle imbalance between multiple muscle groups. They can only use the hip joint and the stride adjustment mechanism frequently to maintain balance. This results in a large increase in body swing for CMT patients [40]. The stepping strategy and the lateral ankle strategy of balance control both need the contribution of the ankle joint. For healthy subjects, the ankle interference torque disturbs the timing, magnitude, and speed of biological torque produced by the ankle joint. For the subject with CMT, the main clinical symptoms, like muscle weakness and atrophy, cause them to have low strength and slow response in their lower limb muscles, which cannot provide the appropriate ankle torque to maintain balance during walking. Although the decrease in walking balance was found in changes in multiple metrics, the decrease in or loss of ankle joint function may be one of the main factors affecting walking balance.

The personal index for each healthy subject could effectively distinguish the state of walking balance under different gait conditions, indicating that the method we proposed is feasible in evaluating human walking balance (Figure 4). A smaller value of the WBI indicates that the subject is more balanced, while a larger value of the WBI indicates that the subject is more unbalanced. The results of the WBI for subject2, shown in Figure 5A, are difficult to distinguish, probably because the effect of a small amount of ankle interference torque is not obvious for healthy subjects who have better balance ability. The means of the WBI in Figure 5B still indicate that our method is effective in evaluating the walking balance, even for a subject with good balance ability.

The general index constructed from the same metrics and weights could still distinguish the state of walking balance for the six subjects (Figure 6). This suggests that our proposed method may be generalized to different individuals and gait conditions. The metrics and weights that were used to construct the general index have certain universality. However, the values of the WBI shown in Figure 6 are different from those shown in Figure 4 because the metrics and weights used in the construction of the WBI are different. This may lead to the loss of some information related to walking balance, but the relative change trends of the index between different gait conditions did not transform. This indicates that the relative rather than the absolute changes of the index are more worthy of consideration.

Not all the preliminary metrics have the ability to evaluate walking balance in our experiments. Some of them showed no significant difference between ZT and MIX conditions (Table 2 and Table 3). Both $COP_{y}$ and $V_{COP_{y}}$ showed strong significant differences in the three time domain characteristics, suggesting that they can well reveal the adjustment strategies for maintaining balance during walking. Relevant studies have shown that the position of the COP in the coronal plane has a certain predictive ability for falling and walking stability [24]. The adjustment strategies of walking balance for different individuals show consistencies and differences. The total number of times there were significant differences in $V_{COP}$, $COP_{x}$, $COM_{z}$, $A_{ANG}$, and MOS for the six subjects was relatively higher (Table 3), indicating that the six subjects made changes in these metrics to maintain their balance. These metrics are more likely to be used as general metrics to construct the general index and evaluate the walking balance of different individuals. Five subjects showed the most frequent significant differences in $V_{COP_{y}}$, but subject6 did not. This result indicates that different subjects have different adjustment strategies to combat ankle interference and maintain walking balance. Certain metrics of the same subject showed significant differences under each gait combination, such as subject2’s $COM_{x}$, subject3’s $COM_{y}$, and subject4’s $COM_{z}$. This may be because the same subject adopted the same walking strategies under different gait conditions, and they did not change their walking strategies because of different external interferences. It is also possibly because the interference was only applied to the ankle joint.

More than 85% of the data information can be represented by five principal components, indicating a great correlation between these metrics (Table 4). From Table 5, it can be seen that the changes in the COP in the direction of the coronal and sagittal axes are closely related to the walking balance of subject1. In addition, we used PCA and analyzed the principal components that contributed more than 10% under ZT and MIX conditions for the other healthy subjects. The $V_{COP}$ and $COP_{y}$ of most of the healthy subjects played an important role in their walking balance, but other metrics were different according to individual differences. This suggests that different individuals have different walking strategies to keep balance. The $COM_{z}$ of subject4 showed significant differences under all gait combinations (Table 3) but only accounted for one item in principal component 4. It was not the most important influencing factor of the walking balance for subject4, which illustrated the importance of PCA in this work. Some balance metrics may be common across different individuals while others not. Therefore, the index calculated by general metrics and weights may have a different effect in evaluating the walking balance of different individuals. If the general metrics and weights used to construct the WBI are not significantly influenced by changes in the walking balance of one subject, it will not lead to an obvious effect on the evaluation of the specific subject’s walking balance. However, the relative change trend of the WBI value is consistent among different walking conditions.

To compare with the existing evaluation methods, the Berg Balance Scale and Tinetti Test were applied after the main experiments for the seven healthy subjects. Results showed that the two scores remained at the maximum for all the healthy subjects even when wearing the unilateral ankle exoskeleton with and without the interference torques. However, subjects said their walking balance was influenced under the interference conditions. We observed that some subjects even changed their walking pattern to prevent themselves from falling. This may be because subjects still had certain ability to maintain walking balance but decreased the state of walking balance when they were walking with interference torques. Such situations cannot be reflected by the scores of Berg Balance Scale or Tinetti Test, but the WBI can reflect the changing of walking balance from the variation trend of its values.

The index constructed by our proposed method could also be used to evaluate the walking balance of the CMT subject, and it was universal between the CMT patient and the healthy subject1 (Figure 7). The state of walking balance for this patient was bad and varied considerably between gait cycles. This result directly reflects the pathological gaits of the patient. The results of the WBI in Figure 7A are different from those in Figure 7B because the metrics and weights used to construct the index were different. To further explore the differences in walking balance between the CMT subject and a healthy subject, we compared the results of the PCA in the two cases (Figure 7A,B). We found that some metrics greatly affected the values of the WBI for the healthy subject and the CMT subject, such as $COM_{z}$, MOS, $V_{COP_{x}}$, and $A_{ANG}$. In the principal components whose total contribution rate was more than 85%, the CMT subject contained more metrics, and some of them did not even appear in the principal components of the healthy subjects, such as COP_CMP. This result indicates that the CMT patient adopted more balance adjustment strategies while walking than the healthy people. Meanwhile, we found that the healthy subject was more inclined to change in the coronal direction, while the CMT subject was more inclined to change in the sagittal direction.

Our findings suggest that a single metric may not be enough to evaluate walking balance. Each balance metric has the ability to assess static and dynamic balance, but it will be changed among different individuals and gait conditions. When human walking balance is influenced, the performance is multi-aspect and personal to different individuals. The variation trend of different metrics reveals the different strategies humans use to maintain walking balance. Personalized customization of the WBI may be more effective during rehabilitation processes. Our work provides a possible method to consider multiple metrics comprehensively in the field of walking balance evaluation. Our proposed method is expected to provide evidence and guidance for walking balance evaluation in future clinical treatment and rehabilitation. The WBI can be used to identify whether the walking balance of human changes on different days and under different gait conditions. The rate of the WBI may be used to predict the changing trend of walking balance and the possibility of falling. By combining Human-in-the-Loop optimization, the WBI can be considered as an objective function of exoskeleton assistance to improve the walking balance of people in need.

There are some possible disadvantages and limitations in this study. The WBI was calculated offline, which means that we could not evaluate the walking balance synchronously when subjects were walking. Since we used the motion capture system and treadmill to collect metrics data, the method is limited to use in the laboratory environment. Our method needs at least two sets of data under relatively balanced and unbalanced gait conditions to construct the WBI. In our experiments, we only applied unilateral ankle interference to the healthy subjects but did not apply multiple external interferences to other parts of the human body. Similar external interference could be added to knee, hip joints, or multiple joints. Our subjects were young people and included only one CMT patient so it is not certain whether this method can be used to evaluate the walking balance of all age groups and different patients. Our proposed method only considers kinematic metrics without revealing the intrinsic physiological mechanism of maintaining walking balance. For future works, we plan to incorporate more physiological and biomechanical metrics into the construction of the Walking Balance Index. Meanwhile, we will recruit more subjects and apply multiple external interferences in future experiments. We hope to apply this method to more people and gait conditions.

## 5. Conclusions

We presented a method to construct a comprehensive evaluation index of human walking balance. We used this method to generate personal and general indexes for different individuals and evaluate the state of their walking balance. We designed walking experiments with exoskeleton interference to collect data from seven healthy subjects and collected data from one CMT subject at multiple speeds. We used statistical analysis to pick the effective metrics from the preliminary metrics and used PCA to construct the index by combining multiple metrics. We not only demonstrated the feasibility and universality of this method in evaluating walking balance for healthy people but also in one patient. The results show consistencies and differences in walking balance adjustment strategies for different individuals under different gait conditions. This study is expected to provide evidence for walking balance evaluation and guidance for future clinical treatment and rehabilitation, improving the walking balance of people in need and allowing further consideration of the WBI as an optimization target of exoskeleton assistance for them in the future.

## Figures and Tables

**Table 1:** Subjects of experiments.

Subject	Height (cm)	Weight (kg)	Peak Torque (N·m)
1	172	53	22
2	170	58	22
3	173	65	22
4	188	90	40
5	175	72	17
6	170	75	15
7	180	62	20
8	160	35.3	-

**Table 2:** Statistical analysis for subject1.

Time Domain Features	*p*-Value						
	Preliminary Metrics						
	${\mathrm{COP}}_{x}$	${\mathrm{COP}}_{y}$	$V_{{\mathrm{COP}}_{x}}$	$V_{{\mathrm{COP}}_{y}}$	MOS	$A_{\mathrm{ANG}}$	COP **_** CMP
Var	0.664	0.000 *	0.002 *	0.000 *	0.000 *	0.002 *	0.081
RMS	0.727	0.000 *	0.006 *	0.000 *	0.041	0.079	0.039
Range	0.144	0.000 *	0.001 *	0.000 *	0.000 *	0.000 *	0.012
	${\mathrm{COM}}_{x}$	${\mathrm{COM}}_{y}$	${\mathrm{COM}}_{z}$	$A_{{\mathrm{COM}}_{x}}$	$A_{{\mathrm{COM}}_{y}}$	$A_{{\mathrm{COM}}_{z}}$	$A_{\mathrm{COM}}$
Var	0.756	0.020	0.000 *	0.107	0.000 *	0.000 *	0.070
RMS	0.001 *	0.011	0.000 *	0.105	0.000 *	0.000 *	0.000 *
Range	0.988	0.004 *	0.000 *	0.058	0.002 *	0.000 *	0.075

* statistically significant difference between ZT and MIX conditions.

**Table 3:** The number of times that the metrics showed significant differences under four gait combinations* for six subjects.

Time Domain Features	Preliminary Metrics				
	${\mathrm{COP}}_{x}$	${\mathrm{COP}}_{y}$	$V_{{\mathrm{COP}}_{x}}$	$V_{{\mathrm{COP}}_{y}}$	COP **_** CMP
Var	4/4/3/2/4/4 **	4/4/4/4/0/4	4/3/2/4/4/2	4/4/4/4/0/4	3/4/2/2/4/1
RMS	0/0/0/0/0/0	0/0/1/1/3/0	4/3/2/4/4/2	4/4/4/4/0/4	0/1/0/1/4/0
Range	4/4/2/2/4/4	4/4/4/4/1/4	4/4/1/4/4/2	4/4/4/4/0/4	2/4/0/2/4/2
	${\mathrm{COM}}_{x}$	${\mathrm{COM}}_{y}$	${\mathrm{COM}}_{z}$	MOS	$A_{\mathrm{ANG}}$
Var	4/4/1/3/4/1	0/4/1/1/0/4	2/4/4/4/4/4	4/4/4/3/2/2	4/3/2/3/4/4
RMS	4/1/1/2/1/1	3/4/1/0/2/0	2/4/4/4/4/4	4/4/0/4/3/0	3/4/2/3/4/4
Range	4/3/1/3/4/2	1/4/1/1/0/4	2/4/4/4/4/4	4/4/4/4/4/2	3/4/2/3/4/4
	$A_{{\mathrm{COM}}_{x}}$	$A_{{\mathrm{COM}}_{y}}$	$A_{{\mathrm{COM}}_{z}}$	$A_{\mathrm{COM}}$	
Var	4/0/1/3/3/3	0/4/4/4/4/0	3/0/1/4/4/4	1/2/1/4/4/4	
RMS	4/0/1/3/3/3	0/4/4/4/4/0	3/0/1/4/4/4	3/0/0/4/4/4	
Range	4/1/2/4/4/3	0/4/4/4/0/2	2/0/0/4/4/4	2/3/1/4/4/2	

* All six subjects completed testing under the zero torque gait condition and four interference torque gait conditions. Four gait combinations were obtained by combining the zero torque condition without interference and four conditions with interference. ** Each element of the table represents the number of times that the metric showed significant differences under the four combinations for subject2/subject3/subject4/subject5/subject6/subject7.

**Table 4:** Principal component analysis for subject1.

Principal Components	Eigenvalue	Cumulative Contribution (%)	Cumulative Contribution (%)
**1**	7.42	**33.74**	33.74
**2**	5.36	**24.36**	58.1
**3**	3.62	**16.43**	74.53
4	1.79	8.14	82.67
5	1.48	6.75	89.42

**Bold font** represents the principal components which contributed more than 10%.

**Table 5:** The coefficient matrix of principal components for subject1.

Time Domain Features	Effective Balance Metrics	Principal Components				
		1	2	3	4	5
Var	$COP_{y}$	**0.330**	−0.075	−0.039	−0.178	−0.157
	$COM_{z}$	0.073	0.257	**0.331**	−0.025	0.110
	$A_{COM_{y}}$	0.278	−0.002	−0.182	0.121	**0.419**
	$A_{COM_{z}}$	0.169	0.265	0.286	0.000	−0.040
	$V_{COP_{x}}$	−0.102	**0.380**	−0.150	−0.073	0.002
	$V_{COP_{y}}$	**0.325**	−0.010	−0.128	−0.145	−0.212
	MOS	−0.047	**0.313**	−0.316	−0.140	−0.088
	$A_{ANG}$	0.122	0.092	−0.118	**0.588**	−0.278
RMS	$A_{COM_{y}}$	0.278	−0.005	−0.179	0.121	**0.425**
	$A_{COM_{z}}$	0.170	0.265	0.285	−0.001	−0.044
	$A_{COM}$	0.203	0.266	0.156	0.081	0.078
	$V_{COP_{x}}$	−0.102	**0.381**	−0.149	−0.072	0.003
	$V_{COP_{y}}$	**0.327**	−0.010	−0.123	−0.147	−0.210
Range	$COP_{y}$	**0.324**	−0.079	−0.004	−0.184	−0.161
	$COM_{y}$	0.245	−0.078	−0.022	−0.170	0.046
	$COM_{z}$	0.073	0.238	0.281	0.033	0.174
	$A_{COM_{y}}$	0.250	0.020	−0.176	0.198	**0.454**
	$A_{COM_{z}}$	0.146	0.168	**0.379**	0.004	−0.095
	$V_{COP_{x}}$	−0.110	**0.346**	−0.248	−0.075	0.005
	$V_{COP_{y}}$	**0.318**	0.002	−0.157	−0.158	−0.204
	MOS	−0.059	**0.317**	−0.311	−0.110	−0.045
	$A_{ANG}$	0.104	0.059	−0.096	**0.606**	−0.326

**Bold font** represents that the coefficient of the metric in this principal component is larger than others.

## Data Availability

The raw data supporting the conclusions of this article will be made available by the authors on request.
